# Simultaneous Voltammetric/Amperometric Determination of Sulfide and Nitrite in Water at BDD Electrode

**DOI:** 10.3390/s150614526

**Published:** 2015-06-19

**Authors:** Anamaria Baciu, Magdalena Ardelean, Aniela Pop, Rodica Pode, Florica Manea

**Affiliations:** Department of Applied Chemistry and Engineering of Inorganic Compounds and Environment, Politehnica University of Timisoara, V. Parvan no. 6, Timișoara 300223, Romania; E-Mails: anamaria.baciu@upt.ro (A.B.); magdalena.ardelean@upt.ro (M.A.); aniela.pop@upt.ro (A.P.); rodica.pode@upt.ro (R.P.)

**Keywords:** simultaneous voltammetric/amperometric detection, nitrite, sulfide, boron-doped diamond electrode, square-wave voltammetry, multiple-pulsed amperometry

## Abstract

This work reported new voltammetric/amperometric-based protocols using a commercial boron-doped diamond (BDD) electrode for simple and fast simultaneous detection of sulfide and nitrite from water. Square-wave voltammetry operated under the optimized working conditions of 0.01 V step potential, 0.5 V modulation amplitude and 10 Hz frequency allowed achieving the best electroanalytical parameters for the simultaneous detection of nitrite and sulfide. For practical *in-field* detection applications, the multiple-pulsed amperometry technique was operated under optimized conditions, *i.e.*, −0.5 V/SCE for a duration of 0.3 s as conditioning step, +0.85 V/SCE for a duration of 3 s that assure the sulfide oxidation and +1.25 V/SCE for a duration of 0.3 s, where the nitrite oxidation occurred, which allowed the simultaneously detection of sulfide and nitrite without interference between them. Good accuracy was found for this protocol in comparison with standardized methods for each anion. Also, no interference effect was found for the cation and anion species, which are common in the water matrix.

## 1. Introduction

Nitrogen-containing and sulfur-containing compounds are ubiquitous in water, and they can be considered as water pollution indexes. Besides nitrate formation, nitrite presence in water occurs by both natural and anthropogenic processes. The natural process consists of the oxidation of nitrogen from the atmosphere by microorganisms in plants, soil and water. Excessive use of fertilizers, as a result of defective management practices in farming activity, inappropriate treatment of wastewater related to the biological step based on nitrification-denitrification processes, or discharges from industrial processes can lead to the presence of nitrogen-containing species exceeding the maximum allowance concentration in water sources [1,2]. The potential hazards of nitrite on human health causing methemoglobinemia or “blue baby” syndrome, stomach cancer by the production of nitrosamines, abortions and central nervous system birth defects are very well-known [3].

Sulfur (II) is an important element from the water point of view due to the fact it is considered one of the major elements in living organisms. Its presence as free sulfide, which includes S^2−^, HS^−^ and H_2_S species, depending on pH, is due to dead organisms and the biological processes which occur particularly in anoxic media [4]. In an anoxic aquatic ecosystem, e.g*.*, seawater, shallow wells, dissolved oxygen is present at low or non-detectable concentration levels and sulphate is reduced to sulfide by anaerobic bacteria [5]. Also, anaerobic reduction of sulfate to sulfide has been adopted as a conventional biological process in municipal wastewater treatment, which represents an important source of sulfide discharged into natural surface waters [6]. In this situation, sulfide is considered a by-product associated with methanogenesis [7]. Various effects of sulfide on human health have been described [8], which requires its quantitative assessment in water.

Since NO_2_^−^ and S^2−^ coexist in aquatic ecosystems, especial in groundwater, and also in surface waters as a result of wastewater discharges, and the exhibit a huge negative impact on human health, the development of a selective and sensitive method for their simultaneous determination is highly desirable.

Maximum allowable concentrations of 0.5 mg·L^−1^ for sulfide and 3 mg·L^−1^ for nitrite are recommended by the World Health Organization [9]. Various analytical methods have been developed for the detection of individual nitrite and simultaneous nitrite and nitrate anions [10,11]. Also, electrochemical methods with very easy and fast operation have been developed in direct relation with the electrode material and the electrochemical techniques [3,12].

For the quantitative assessment of sulfide, several analytical methods have been reported [13,14]. Most of the electrochemical methods reported are based on potentiometic principles using ion-selective sensors [15], and there are a few voltammetric/amperometric techniques [16,17,18,19].

In recent years, many efforts have been directed to the enhancement of the performance of voltammetric and amperometric techniques. Differential pulse voltammetry (DPV) and square wave voltammetry (SWV) are very efficient for electroanalysis applications due to their high sensitivity, based mainly on background current minimization. These techniques belong to the differential multipulse techniques in according to the literature classification [19], which allows reaching an enhanced useful current signal (ΔI) corresponding to a more evidenced current peak recorded after current sampling at the end of the consecutive pulses by plotting current difference versus potential. Multiple-pulsed amperometry (MPA) can be regarded as an alternative to chronoamperometry to avoid the electrode fouling by *in-situ* electrochemical cleaning and also, to develop protocols for the simultaneous amperometric detection [3].

Boron-doped diamond (BDD) electrodes are a very popular electrode applied in many electroanalytical applications [20,21] due to its remarkable peculiarities related to the large potential window and very low background current, which represent very important characteristics for electroanalysis.

To our knowledge, there are no reports about the simultaneous detection of sulfide and nitrite in aqueous matrices. The aim of our study was thus to develop electroanalytical strategies based on certain voltammetric and amperometric techniques for the simultaneous and sensitive detection of sulfide and nitrite anions in aqueous solution using a boron-doped diamond (BDD) electrode. Direct oxidation and determination of sulfide and nitrite were investigated by cyclic voltammetry (CV), square-wave (SWV) and differential-pulsed voltammetry (DPV) techniques. Chronoamperometry (CA) was tested for simultaneous detection and multiple-pulsed amperometry (MPA)-based procedures were developed and optimized for selective and simultaneous detection of sulfide and nitrite.

## 2. Experimental Section

All electrochemical measurements were carried out using an Autolab potentiostat/galvanostat PGSTAT 302 (Eco Chemie, Utrecht, The Netherlands) controlled with the GPES 4.9 software and a three-electrode cell, with a saturated calomel electrode as reference electrode, a platinum counterelectrode and commercial BDD working electrode. A BDD working disc electrode with 3 mm diameter characterized by a doping degree of about 0.1% boron was purchased from Windsor Scientific, Slough Berkshire, UK. The electrochemical behaviour of the electrode envisaging nitrite and sulfide detection was studied by cyclic voltammetry (CV), differential-pulsed voltammetry (DPV), square-wave voltammetry (SWV), chronoamperometry (CA) and multiple-pulsed amperometry (MPA). Before each electrochemical experiment, three repetitive cyclings between −0.5 V and +1.25 V *vs.* SCE in 0.1 M Na_2_SO_4_ supporting electrolyte were performed as an electrochemical pre-treatment. Sodium nitrite, sodium sulfide and sodium sulfate were purchased from Merck (Darmstadt, Germany), as analytical grade reagents and the solutions were freshly prepared with doubly distilled water.

To improve the electroanalytical response of the BDD electrode for the detection of nitrite and sulfide, expressed as useful signal (ΔI) that represents the current readed at the detection potential from which the background current is substracted, differential-pulsed voltammetry (DPV) and square-wave voltammetry (SWV) techniques were exploited under various operating conditions for optimization. The modulation amplitude (a), step potential (ΔEs) and frequency (f) were varied in order to achieve the best sensitivity and the lowest limit of detection (LOD), calculated as three times the standard deviation for the blank solution divided by the slope of the calibration plots. For the nitrite detection in the presence of sulfide, to determine the slope of the calibration plots that assesses the sensitivity, the background current means the current recorded in the presence of sulfide, which is substracted from the current recorded in the presence of nitrite at a certain concentration.

## 3. Results and Discussion

### 3.1. Voltammetric Studies

**Figure 1:**
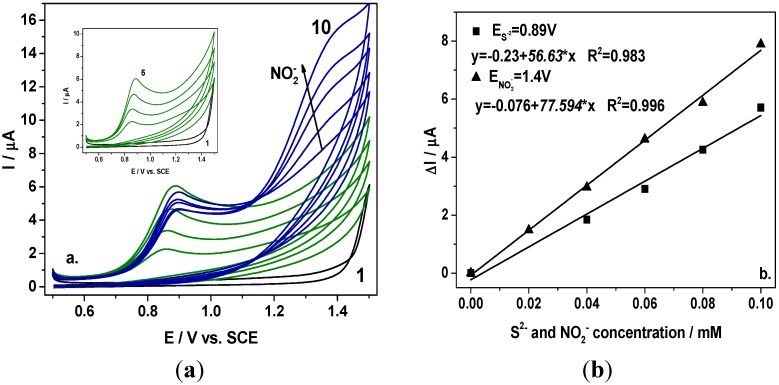
(**a**) Cyclic voltammograms recorded at a BDD electrode in 0.1 M Na_2_SO_4_ supporting electrolyte (curve 1) in the presence of 0.02–0.1 mM sulfide (curves 2–5; Inset of figure), and 0.02–0.1 mM nitrite (curves 6–10) at a potential scan rate of 0.05 Vs^−1^ in a potential range from +0.5 to +1.5 V/SCE; (**b**) Calibration plots of the current *vs*. anion concentration recorded at E = +0.89 V *vs*. SCE for sulfide and E = +1.4 V *vs.* SCE for nitrite.

Taking into account the electrochemical behaviour of the BDD electrode in the presence of sulfide and nitrite alone in 0.1 M Na_2_SO_4_ supporting electrolyte (results not shown here) in relation with the oxidation potential value (+0.9 V/SCE for S^2−^ and +1.4 V/SCE for NO_2_^−^) and the sensitivity (55 µ·Am·M^−1^ for S^2−^ and 67 µ·Am·M^−1^ for NO_2_^−^), the question that was raised is whether nitrite can be detected in the presence of sulfide envisaging their simultaneous detection. In Figure 1 are presented the cyclic voltammograms recorded at the BDD electrode in the presence of sulfide within the concentration range from 0.02 mM to 0.1 mM and further, after adding nitrite within the same concentration range. It can be noticed that nitrite oxidation started at +1.15 V/SCE and the oxidation peaks are well-separated for each anion. In comparison with the results obtained for individual detection of nitrite, the presence of sulfide led to a shifting of the potential value at which the nitrite oxidation started to a more positive potential. However, the detection potential value was not influenced, and the nitrite detection was recorded at +1.4 V/SCE.

A linear dependence between oxidation peak height and anion concentration was found with good correlation coefficient. The sensitivities determined under these conditions were almost similarly with those obtained for the detection of individual anion. It should be mentioned that within the framework of this scheme, the sulfide is detected individually and nitrite is detected in the presence of sulfide.

**Figure 2:**
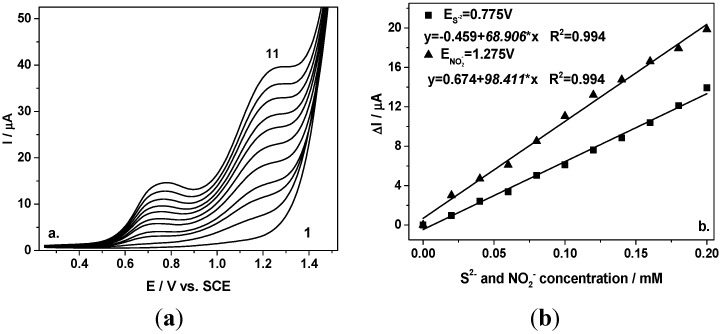
(**a**) Differential-pulsed voltammograms recorded at the BDD electrode in 0.1 M Na_2_SO_4_ supporting electrolyte (curve 1) in a mixture of 0.02–0.2 mM sulfide and nitrite (curves 2–11), under 0.2 V modulation amplitude, 0.01 V step potential, and 0.1 V·s^−1^ scan rate in a potential range from +0.25 to +1.5 V/SCE; (**b**) Calibration plot of the current *vs*. anions concentration recorded at E= +0.775 V *vs*. SCE for sulfide, respectively, and at E = +1.275 V *vs*. SCE for nitrite.

These results are very promising and further experiments were conducted to improve the sensitivity for simultaneous voltammetric detection of nitrite and sulfide. Thus, the differential-pulsed technique (DPV) was applied under the operating conditions of 0.2 V modulation amplitude and 0.01 V step potential by alternative adding of each sulfide and nitrite species. Figure 2a depicts the DPVs recorded with the BDD electrode under the conditions presented above. Linear dependences of peak current versus sulfide and nitrite concentration, respectively, with good correlation coefficients were achieved.

In comparison with CV, the electroanalytical performance related to the sensitivity and the detection potential was improved. Better sensitivity was achieved for both anions, 68.9 *vs.* 56.63 µ·Am·M^−1^ for S^2−^ and 98.41 *vs.* 77.59 µ·Am·M^−1^ for NO_2_^−^. Also, the detection potentials were shifted to less positive potential, maintaining the same about 500 mV potential separation between the two detection potentials.

In order to further improve the electroanalytical performance of BDD for simultaneous detection of sulfide and nitrite, the square-wave voltammetry technique (SWV) was applied under various operating conditions in order to optimize the results with the best sensitivities and detection potential. A similar working protocol to that described previously was used for the simultaneous detection of both anions. An optimization procedure was performed under the operating conditions gathered in Table 1, which also presents the sensitivity and the detection potential for each anion. 

**Figure 3:**
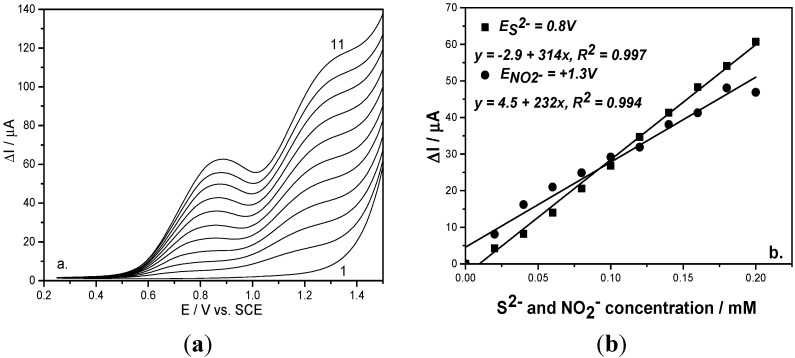
(**a**) Square-wave voltammograms recorded on a BDD electrode under 0.01 V step potential and 0.2 V modulation amplitude, 10 Hz frequency, 0.1 V·s^−1^ scan rate, between −0.25 and +1.5 V *vs*. SCE in 0.1 M Na_2_SO_4_ supporting electrolyte (curve 1) and in the presence of mixtures of 0.02–0.2 mM sulfide and nitrite concentrations (curves 2–11); (**b**) Calibration plots of the current *vs*. anion concentrations recorded at: E = +0.8 V/SCE for sulfide concentration and E = +1.3 V/SCE for nitrite concentration.

**Figure 4:**
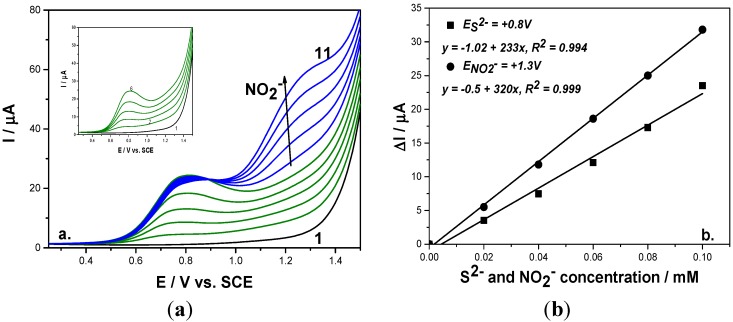
(**a**) Square-wave voltammograms recorded on a BDD electrode under 0.01 V step potential and 0.2 V modulation amplitude, 10 Hz frequency, scan rate 0.1 V·s^−1^, between −0.25 and +1.5 V *vs*. SCE in 0.1 M Na_2_SO_4_ supporting electrolyte (curve 1) in the presence of 0.02–0.1 mM mixtures of sulfide (curves 2–6; Inset of figure), and 0.02–0.1 mM nitrite (curves 7–11). (**b**) Calibration plots of the current *vs*. anion concentration recorded at: E = 0.8 V/SCE *vs*. sulfide concentration and E = +1.3 V/SCE *vs*. nitrite concentration.

To check the reproducibility and the interference of sulfide on nitrite detection, two working protocols related to anion addition were proposed. The first representing the detection of nitrite in the presence of sulfide consisted of adding a sulfide concentration up to desired maximum level followed by adding nitrite concentrations (Figure 3a). The second working protocol consisted of alternative additions of each anion (Figure 4a). For each working protocol, the linear calibration plots of current *versus* each anion concentration showed good correlation coefficients (Figure 3b and Figure 4b). Based on these results, it can be observed that the sensitivities for each anion under both working protocols were similar, which shows that the results are reproducible.

**Table 1:** Operating conditions for SWV and electroanalytical performance recorded at BDD electrode for simultaneous detection of sulfide and nitrite.

a (V)	ΔE_s_ (V)	f (Hz)	Anion	E_ox_ (V/SCE)	Sensitivity (µA/mM)	*R* ^2^
0.01	0.001	50	S^2−^	0.92	11.4	0.996
			NO_2_^−^	1.355	7.7	0.975
		100	S^2−^	0.918	14.6	0.998
			NO_2_^−^	1.385	11.0	0.970
0.1	0.01	10	S^2−^	0.85	150.4	0.997
			NO_2_^−^	1.35	135.7	0.992
0.2	0.01	10	S^2−^	0.8	**314.0**	0.998
			NO_2_^−^	1.3	**232.0**	0.993
	0.02	10	S^2−^	0.805	280.4	0.990
			NO_2_^−^	1.3	183.7	0.966
0.5	0.01	10	S^2−^	0.63	292.0	0.997
			NO_2_^−^	1.02	198.0	0.973
	0.02	10	S^2−^	0.63	**396.0**	0.997
			NO_2_^−^	1.02	**300.0**	0.981

In relation with the best sensitivity and less positive detection potential values, the optimum operating conditions for SWV were determined, *i.e.*, 0.5 V modulation amplitude (a), 0.02 V step potential (ΔE_s_) and 10 Hz frequency (f). Also, comparable results in relation with the sensitivity were achieved for 0.2 V modulation amplitude, 0.01 V step potential and 10 Hz frequency and the difference consisted of a more positive detection potential value. However, it must be mentioned that the better potential separation was reached for these last operating conditions. The choice of operating conditions will be based on the concrete objective related to the final application. In comparison with the voltammetric techniques presented above, CV and DPV, the SWV technique allowed the best analytical performance for simultaneous sulfide and nitrite detection (see Table 1).

### 3.2. Amperometric Studies

It is well-known that chronoamperometry is the easiest electrochemical technique for detection applications. Having CV results as a reference basis, the operating conditions for CA were set up to detect each anion by applying two potential levels characteristic of the detection potential corresponding to sulfide (+0.85 V/SCE) and nitrite (+1.25 V/SCE) oxidation. It should be mentioned that under CA conditions, by applying the second detection potential level of +1.4 V/SCE no reproducible results were obtained. A possible reason for this situation could be the initiation of oxygen evolution that overlapped with the oxidation process. The current corresponding to the oxygen evolution cannot be controlled and thus, no reproducible results were achieved at the potential value of +1.4 V/SCE.

**Figure 5:**
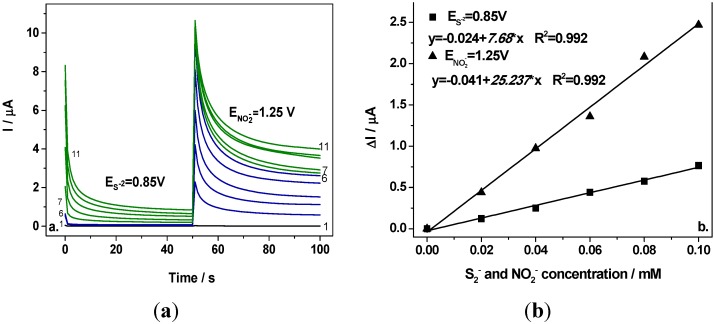
(**a**) Chronoamperograms recorded at BDD electrode in 0.1 M Na_2_SO_4_ supporting electrolyte (curve 1) in the presence of 0.02–0.1 mM nitrite (curves 2–6) in a mixture of 0.02–0.1 mM sulfide and 0.1 mM nitrite (curves 7–11), at the potential values of E_1_ = +0.85 V/SCE for sulfide and E_2_ = +1.25 V/SCE for nitrite; (**b**) Calibration plot of the current *vs*. anion concentration recorded at E_1_ = +0.85 V/SCE for sulfide (curves 7–11), respectively E_2_ = +1.25 V/SCE (curves 2–6) for nitrite.

Figure 5a shows two-level chronoamperograms recorded at the first potential level of +0.85 V/SCE and the second potential level of +1.25 V/SCE in 0.1 M Na_2_SO_4_ supporting electrolyte (curve 1) and in the presence of various nitrite concentrations (curves 2–6) and the mixture of 0.1 mM nitrite and various concentrations of sulfide (curves 7–11). It can be easily noticed that at the first potential level corresponding to the sulfide oxidation no signal corresponding to nitrite detection was recorded (curves 2–6 at E = +0.85 V/SCE) and the signal was recorded only by adding sulfide, which increased linearly with sulfide concentration (see Figure 5b, curve 1). At the second potential level operated at E = +1.25 V/SCE, the useful currents recorded after running for 100 s (Figure 5a, curves 2–6) depended linearly on the nitrite concentration (see Figure 5b, curve 2). For each anion detection a good correlation coefficient was achieved. The sensitivities determined under CA operating conditions were lower in comparison with CV, which could be explained by a possible electrode fouling that is characteristic when applying chronoamperometry by current measurements at the detection potential for long time. Analyte and the oxidation products should be responsible for the electrode fouling. Moreover, the presence of sulfide is detected also, at the potential level corresponding to nitrite detection (+1.25 V/SCE) that means that sulfide should interfere with nitrite detection. At this potential level, a cumulative signal corresponding to the presence of the sum of both anions will be detected. The main problem in this situation is that the useful current corresponding to the presence of sulfide did not depend linearly on the sulfide concentration at this potential level and no quantitative assessment of nitrite can be achieved under these conditions.

In order to simultaneously detect each anion without the interference of the other as an amperometric detector, the multiple-pulsed amperometry technique was explored and the detection scheme was elaborated based on the potential levels number, values and operating time. In the first stage, the multiple-pulsed amperometry technique was operated under similar conditions to the CA and even if for nitrite the sensitivity was better, no signal for sulfide detection was found (results are not shown here). Under these conditions, the reference points for the potential values for three levels based multiple-pulsed amperometry applying were selected based on the curve CV shape. The two detection potential values were chosen based on the oxidation potential value for each anion, *i.e*., +0.85 V /SCE for sulfide and +1.25 V/SCE for nitrite, the last one being considered also as a cleaning step to avoid fouling the electrode and the third one maintained at −0.5 V/SCE as a conditioning step. This scheme was applied for the continuous addition of nitrite for concentrations ranging from 0.02 mM to 0.1 mM followed by continuous addition of sulfide at the same concentration range to be able to follow the signals recorded for each anion.

**Figure 6:**
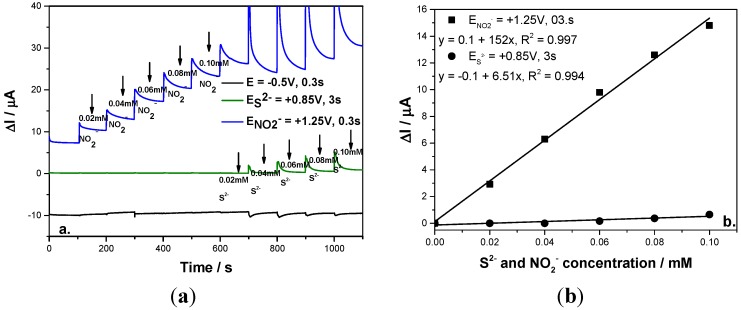
(**a**) Multiple-pulsed amperograms recorded at a BDD electrode in 0.1 M Na_2_SO_4_ supporting electrolyte and adding consecutively and continuously each of 0.02 mM nitrite and respective, sulfide, recorded at E_1_ = −0.5 V/SCE, E_2_ = +0.85 V/SCE, E_3_ = +1.25 V/SCE; (**b**) Calibration plot of the current *vs*. anion concentration recorded at: E_2_ = +0.85 V/SCE *vs*. sulfide concentration and E_3_ = +1.3 V/SCE *vs*. nitrite concentration.

To avoid the interference of sulfide in nitrite detection, a very important parameter in this scheme was found to be the time at which each potential level is maintained. This parameter was optimized in relation with the time allocated to the oxidation of both anions. It was found that by applying the same time for sulfide and nitrite oxidation, only the signal for nitrite detection was recorded and sulfide was not detected, probably due to the fact the sulfide oxidation process is slower in comparison with the nitrite oxidation process in terms of the mass transport. By increasing the time corresponding to the sulfide oxidation to assure a 10:1:1 ratio between sulfide oxidation time:nitrite oxidation time:cleaning time, sulfide detection was possible with the similar sensitivity to that achieved by the CA technique and no influence on nitrite detection was found by applying the above-described MPA (see Figure 6a). The concentration step for both anions was 0.02 mM that was added continuously into the supporting electrolyte and both concentrations ranged from 0.02 mM to 0.1 mM. Moreover, the sensitivity for the nitrite detection was much improved in comparison with CA (Figure 6b).

**Figure 7:**
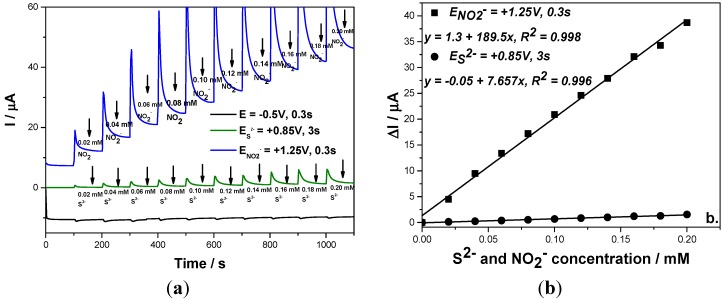
(**a**) Multiple-pulsed amperograms recorded at a BDD electrode in 0.1 M Na_2_SO_4_ supporting electrolyte and adding continuously a mixture of 0.02 mM NO_2_^−^ and 0.02 mM S^2−^, recorded at E_1_ = −0.5 V/SCE, E_2_ = +0.85 V/SCE, E_3_ = +1.25 V/SCE; (**b**) Calibration plot of the current *vs*. anion concentration recorded at: E_2_ = +0.85 V/SCE *vs*. sulfide concentration and E_3_ = +1.3 V/SCE *vs*. nitrite concentration.

This detection scheme was also applied for the simultaneous amperometric detection of both anions in their mixtures and the results were reproducible (Figure 7). Based on these results, it can be concluded that the simultaneous detection of sulfide and nitrite without interference with each other to other was achieved using a three levels-MPA detection procedure for which the pulses were applied continuously based on the following scheme:

(a) −0.5 V/SCE for a duration of 0.3 s, as conditioning step.
(b) +0.85 V/SCE for a duration of 3 s, which assure the sulfide oxidation.
(c) +1.25 V/SCE for a duration of 0.3 s, which assure the nitrite oxidation.

The electroanalytical parameters established for the simultaneous detection of sulfide and nitrite under the optimum operating conditions for each voltammetric/amperometric technique used are gathered in Table 2. It can be noticed that the best electroanalytical parameters for the simultaneous voltammetric detection were acheived by the SWV technique, while for the simultaneous amperometric detection the three levels-MPA technique led to very good results, this technique being more suitable for *in-field* practical applications.

**Table 2:** The electroanalytical parameters of simultaneous detection of voltammetric/amperometric detection of sulfide and nitrite at a BDD electrode using different techniques.

Tehnique	E,V/SCE		Sensitivity, µA/mM		Correlation Coefficient, R^2^		LOD, mM	
	S^2−^	NO_2_^−^	S^2−^	NO_2_^−^	S^2−^	NO_2_^−^	S^2−^	NO_2_^−^
CV	0.9	1.35	56.63	77.94	0.998	0.994	8.66 × 10^−4^	2.88 × 10^−3^
DPV	0.77	1.27	68.90	12.75	0.997	0.995	1.83 × 10^−4^	6.09 × 10^−4^
SWV/ Step potential: 0.01 V; Modulation amplitude: 0.2 V; Frequency: 10 Hz	0.90	1.3	314	232	0.997	0.987	5.55 × 10^−5^	1.84 × 10^−4^
SWV/ Step potential: 0.01 V; Modulation amplitude: 0.5 V; Frequency: 10 Hz	0.66	1.04	396	300	0.997	0.981	1.16 × 10^−5^	3.86 × 10^−4^
CA	0.85	1.25	7.68	25.23 *	0.992	0.992	9.83 × 10^−4^	7.14 × 10^−3^
MPA	0.85	1.25	7.66	189	0.996	0.998	7.17 × 10^−4^	2.39 × 10^−3^

* for this sensitivity there is a contribution of sulfide.

A recovery test was performed by analyzing three parallel tapwater samples, which contained 0.05 mM sulfide and 0.05 mM nitrite. This test was run in 0.1 M Na_2_SO_4_ as supporting electrolyte and a recovery of 94% with a relative standard deviation of 3.8% was found for sulfide and a recovery of 96% with a relative standard deviation of 2.8% was found for nitrite using the three potentials-MPA procedure presented above. Finally, the results obtained by this method were compared with those obtained by means of the standardized methods for nitrite and sulfide determination [22]. Based on the results obtained, it can be concluded that the two methods lead to very close results and that the accuracy of the proposed MPA method is good. The interference effect of various anion and cation species that are common water matrix components was investigated in the presence of 0.1 mM Na_2_S and 0.1 mM NaNO_2_. These species were added at the concentrations 100 times higher. No interference effect was noticed in the presence of Mg^2+^, Ca^2+^, Mn^2+^, Fe^3+^, SO_4_^2−^, NO_3_^−^, CO_3_^2−^, I^−^, Cl^−^, F^−^.

## 4. Conclusions

This work presented new protocols for the simultaneous detection of sulfide and nitrite in aqueous solutions using cyclic voltammetry, differential-pulsed voltammetry, square-wave voltammetry and multiple-pulsed amperometry techniques at a commercial BDD electrode. The chronoamperometry technique could not substantially differentiate NO_2_^−^ and S^2−^ and a cumulative signal corresponding to both anions was recorded at the characteristic potential value of NO_2_^−^ detection, which indicated an interference effect. Multiple-pulsed amperometry technique operated under optimized conditions, *i.e.*, −0.5 V/SCE for a duration of 0.3 s as conditioning step, +0.85 V/SCE for a duration of 3 s that assures the sulfide oxidation and +1.25 V/SCE for a duration of 0.3 s, where the nitrite oxidation occurred, allowing the simultaneous detection of sulfide and nitrite without interfering with each other. By comparing the MPA results with those obtained by standardized methods for each anion, it was found that this MPA based method is characterized by a good accuracy.

The best electroanalytical parameters for the simultaneous detection of nitrite and sulfide were achieved by square-wave voltammetry operated under the optimized operating conditions consisting of a step potential of 0.01 V, modulation amplitude of 0.5 V and frequency of 10 Hz. Also, the modulation amplitude of 0.2 V led to the best performance regarding the lowest limit of detection for nitrite detection.

Based on the results regarding the detection performance in relation with the sensitivity, the lowest limit of detection, the accuracy and the interference effect, the elaborated protocol that involve BDD electrode exhibits a great potential for the practical applications. Even if the voltammetric technique exhibited better electroanalytical performance *versus* the multiple-pulsed amperometric technique, the selection of the technique will be in relation with specific practical application, *i.e*., *in-vitro* or *in-field* detection practical applications.
